# Synergistic effect of FOXM1 and BCL-2 inhibition in a preclinical treatment study on multiple myeloma

**DOI:** 10.1186/s12967-024-05452-9

**Published:** 2024-07-08

**Authors:** Vivian Zhou, Manya Yu, Jiaqi Fu, Siegfried Janz, Xing Cui

**Affiliations:** 1 Department of Medicine, Cancer Center-Froedtert Hospital, Medical College of Wisconsin, Milwaukee, WI 53226 USA; 2 The First School of Clinical Medicine, Shandong University of Traditional Chinese Medicine, Jinan, 250001 Shandong Province, PRC China; 3 Department of Oncology and Hematology, The Second Affiliated Hospital of Shandong University of Traditional Chinese Medicine, Jinan, 250001 Shandong Province, PRC China

To the editor:

Multiple myeloma (MM) is a hematologic malignancy that remains incurable despite treatment advances. Many patients experience relapse or are refractory to existing therapies. There are no standard guidelines for the care of relapsed/refractory MM (RRMM). Emerging treatment options include CAR-T cell therapy and bispecific antibodies. For patients carrying t(11;14) BCL-2 inhibitors, it is sometimes recommended to use venetoclax alone or in combination. FOXM1 is a central transcription factor within the FOX family, influencing cell proliferation and cell cycles [1]. Its aberrant expression correlates with multiple cancers [2]. Further studies show its implications for cancer therapies [3]. FOXM1 is also a promising target in high-risk MM and RRMM, driving myeloma metabolism and drug sensitivity [4]. The *Wen* group at Marshfield Clinic Research Institute confirmed that inhibiting FOXM1 with NB73 increases the sensitivity of myeloma cells to venetoclax in vitro [5]. Here, we conducted in vivo and ex vivo experiments to further confirm the synergistic effect of NB73 and venetoclax in the treatment of MM.

We transplanted OPM2 cells stably expressing Renilla Luciferase, a reporter for in vivo imaging, into NOD.Cg Prkdcscid Il2rgtm1Wjl/SzJ (NSG) mice via tail vein injection. Three weeks later, we assessed the engraftment of OPM2 cells in each NSG mouse using in vivo imaging after injecting Renilla Luciferase substrate and measuring bioluminescence. A substantial emission of bioluminescence indicated successful engraftment of OPM2 cells in NSG mice (Fig. 1A). Mice with successful engraftments were then divided into Vehicle, NB73, Venetoclax, and the NB73-Venetoclax combination groups (*n* = 7/group), with each group showing equal levels of bioluminescence, indicating similar MM burden among the four groups (Fig. 1A). Survival of NSG mice was monitored following the start of treatments. Compared to the Vehicle control, NB73 alone and Venetoclax alone led to mild increases in mouse survival, although the increases were not statistically significant (Fig. 1B). However, the NB73-Venetoclax combination significantly extended the survival of NSG mice, indicating that the combination effectively delayed disease progression in vivo (*p* < 0.0036, Fig. 1B). Of note, the dose of Venetoclax was administrated as 100 mg/kg, three times weekly, which was not high compared to 400 mg/kg/day. Our data highlight the potential of combining a FOXM1 inhibitor and a BCL2 inhibitor for the treatment of MM.

**Fig. 1:**
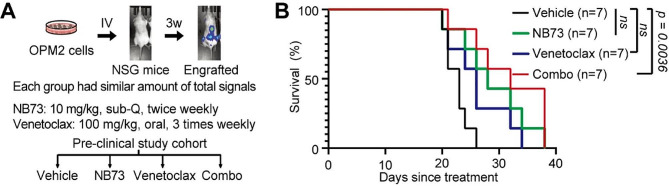
Inhibiting FOXM1 and BCL2 prolongs the survival of NSG mice engrafted with OPM2 cells. (**A**) The experiment involved transplanting OPM2 cells expressing Renilla Luciferase into NSG mice via tail vein. Successful engraftment was confirmed using IVIS imaging at the third week. Mice were then divided into four groups based on similar Luciferase activities and treated with NB73, Venetoclax, or their combination as indicated. (**B**) Survival curves show that inhibiting FOXM1 and BCL2 prolonged the survival of NSG mice engrafted with OPM2 cells compared to controls. Statistical analysis using Logrank test with Bonferroni correction for multiple comparisons was performed to calculate p values

Multi-omics assays and drug sensitivity tests play crucial roles in guiding personalized cancer therapies. The rapid development of multi-omics tools has made themselves easily accessible in both university and community hospitals. However, drug sensitivity testing remains a significant challenge for most community hospitals, contributing to the inferior outcomes of MM in under-represented populations. Testing primary MM cells requires academic settings with well-trained researchers and costly devices to perform the complicated ex vivo culture protocol. These challenges have forced community hospitals to ship patient specimens to university hospitals, inevitably causing substantial cell death during shipment. Therefore, we developed the protocol of enriching the primary MM cells using anti-human CD138 antibody and validated the simplified ex vivo culture protocol designed by the *Wen* group that can be implemented by a technician with regular cell culture settings in a community hospital (Fig. 2A). In brief, mononuclear cells were harvested from bone marrow specimens using density gradient centrifugation. After enrichment with magnetic beads conjugated with anti-CD138 antibody, MM cells were cultured in RPMI-1640 medium with 20% heat-inactivated autologous serum at 37°C and 5% CO_2_. Compared to the cells at the 0 h, the cells at the 18th hour in cell incubators retained 70% of cell viability (Fig. 2B), indicating that the majority of MM cells remained viable after 18 h of ex vivo culturing.

**Fig. 2:**
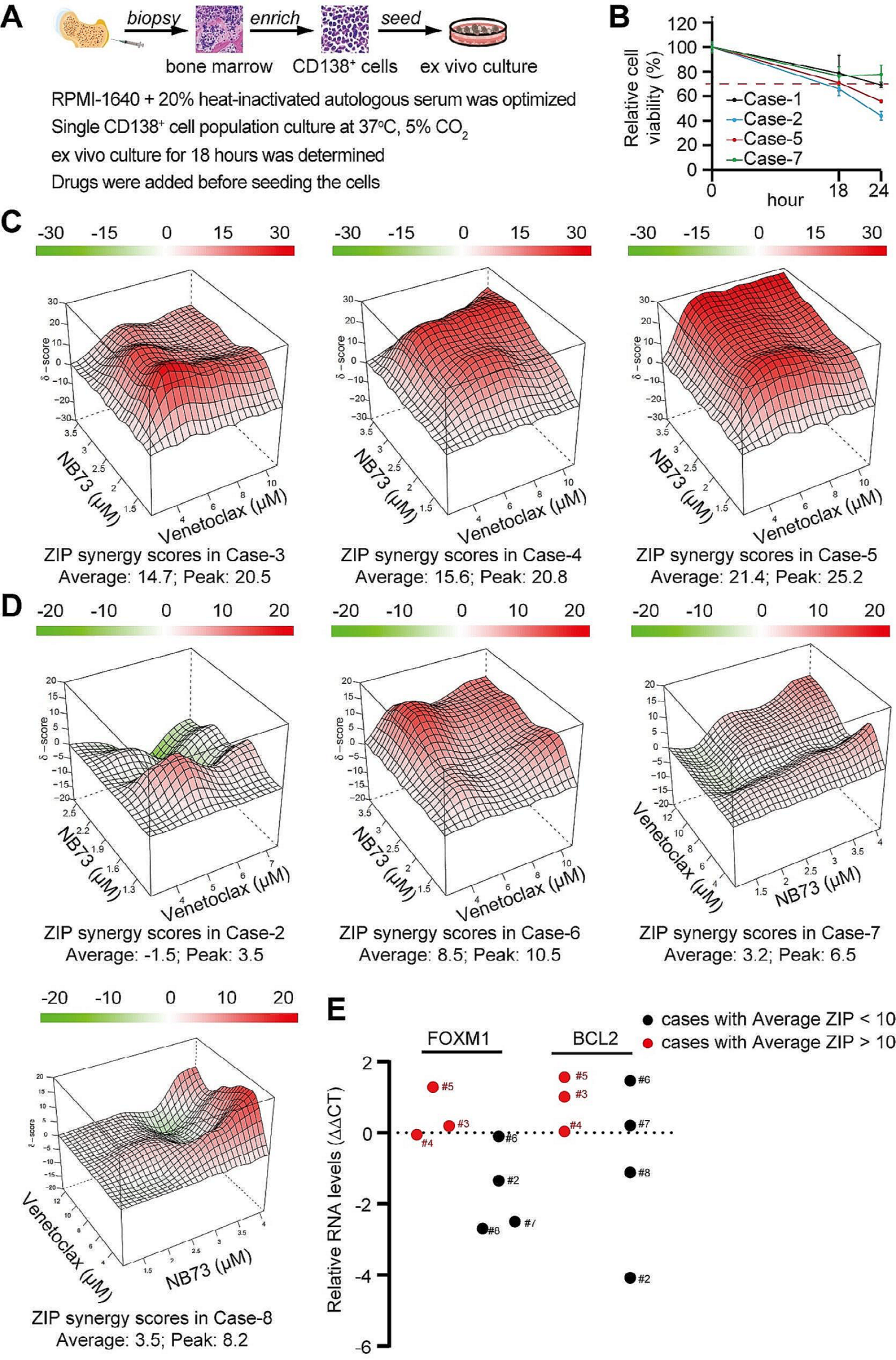
A simplified ex vivo culture system was developed to evaluate the synergy between NB73 and Venetoclax in primary multiple myeloma cells. (**A**) Illustration of simplified ex vivo culture for primary MM cells without cytokine supplementation. (**B**) After 18 h, primary MM cell viability remained at 70% compared to baseline. (**C**) Drug synergy between NB73 and Venetoclax was observed in three cases post 18-hour ex vivo culture. (**D**) No observed drug synergy between NB73 and Venetoclax in three cases following 18-hour ex vivo culture. (**E**) Relative RNA levels of FOXM1 and BCL2 were assessed using qRT-PCR in the seven cases

Consistent with the in vitro study by Zhi et al., a recent 24-hour drug screening was conducted using primary MM cells with RPMI-1640 medium plus 10% FBS in a co-culture setting by *Kropivsek* group. We applied our simplified ex vivo protocol to conduct the ZIP drug synergy assay of NB73 and Venetoclax in seven MM cases (supplemental Table-S1). The synergy of NB73 and Venetoclax was observed in three cases but not in the other four cases (Fig. 2C-D). We examined the expressions of FOXM1 and BCL2 in MM cells from these seven cases using qRT-PCR assay. We detected higher expressions of FOXM1 and BCL2 in the MM cells where the drug synergy was observed than in the MM cells where the drug synergy was not observed (Fig. 2E), implying that these MM cells with high FOXM1 and BCL2 signaling are more sensitive to FOXM1 inhibitor and BCL2 inhibitor.

Overall, based on previous in vitro experiments, we further demonstrated the synergistic effect of FOXM1 inhibitor and venetoclax in vivo and ex vivo. This study provides evidence for revealing the important role of FOXM1 in MM and the combination therapy in MM. Undeniably, this study has several limitations. The first is that the limited number of cases; hence, future studies should focus on expanding the sample size. Moreover, this study is primarily confined to phenotypic research. Therefore, further investigations are essential to delve into the mechanism underlying the synergistic effect between FOXM1 and venetoclax.

### Electronic supplementary material

Below is the link to the electronic supplementary material.

Supplementary Material 1

Supplementary Material 2

## Data Availability

The datasets during and/or analysed during the current study available from the corresponding author on reasonable request.
