# Supplementary material for: Synergistic effect of FOXM1 and BCL-2 inhibition in a preclinical treatment study on multiple myeloma
Source: J Transl Med. 2024 Jul 8;22:638. doi: 10.1186/s12967-024-05452-9 (PMC11232139; doi:10.1186/s12967-024-05452-9)
Supplement: Supplementary file 1 — Supplementary Material 1 [file 12967_2024_5452_MOESM1_ESM.docx]

**Materials and Methods:**

**Cell lines**

OPM2 cell line was authenticated in Dr. Janz’s lab. FBS (GIBCO, cat# 16000069) was heat-inactivated at 56°C for 30 minutes and added to RPMI-1640 medium (ATCC, GIBCO & HYCLONE) to a final 10% concentration. All cell lines were cultured in petri dishes at 37°C with 5% CO2 and passaged every two or three days.

**Drugs and chemicals**

Venetoclax was obtained from TargetMol (Wellesley Hills, MA) and dissolved in DMSO at 10 mM. NB73, developed by Drs. Katzenellenbogen, was first dissolved in ethanol at 10 mM, and then subsequently diluted to 5 mM in DMSO.

**Ex vivo culture protocol**

The bone marrow specimens were transported to the lab within 2 hours after biopsy. On average, it took 2-3 hours from receiving cells to having MM cells in the cell incubator per sample. The drug treatment duration was 18 hours. The completion of the CellTiter Glo assay took approximately 20 minutes per 96-well plate. Data processing using the Excel program and free online ZIP drug synergy scoring program took about 1 hour. Overall, our protocol allows for the drug sensitivity data to be ready for clinicians' consideration within 24 hours. Detailed Protocol as below:

1. Mix bone marrow specimens (20–30 mL) with an equal volume of room temperature PBS.
2. Slowly layer 6 mL of the bone marrow-PBS mixture on top of 7.5 mL of Lymphocyte Separation Solution (TBD Science, cat# LTS1077) in a 15-mL cone-bottom centrifuge tube. Do not mix and keep at room temperature.
3. Centrifuge at 500×g for 25 minutes at 20°C. The lymphocyte layer forms the second layer from the top. Collect lymphocytes (1-2 mL cells per tube) without disturbing the first layer. Mix in a new 50-mL centrifuge tube and count live cells with trypan blue staining.
4. Centrifuge cells at 300×g for 10 minutes at 20°C and discard supernatant.
5. Resuspend cell pellet in 80 µL PBS per 2x107 total cells and transfer to a new 15-mL tube on ice (exactly 2x10^7^ cells in 80 µL).
6. Add 20 µL of cold microbeads conjugated with anti-human CD138 antibody (Miltenyi, cat# 130-051-301) to 2x10^7^ cells.
7. Mix well by pipetting on ice and incubate for 15 minutes in the dark.
8. Wash cells with 1-2 mL cold PBS per 2x10^7^ cells by pipetting gently.
9. Centrifuge cells at 300×g for 10 minutes at 4°C.
10. Resuspend cells in 500 µL cold 0.5% BSA in PBS (Sparkjade, cat# ED0017-B) at a concentration of 108 cells per 500 µL.
11. Pre-rinse MACS columns with 1 mL room temperature PBS.
12. Load cells-microbeads mixture onto the column.
13. Wash column with 500 µL room temperature PBS three times after the cell solution goes through the column completely.
14. Remove column from separator to a new 15-mL collection tube.
15. Add 1 mL cold 0.5% BSA in PBS onto column and immediately flush cells into collection tube within 1-2 seconds.
16. Elute column three times to obtain 3 mL cell suspension.
17. Count collected cells with trypan blue staining (>95% live cells).
18. Centrifuge 8-10 mL peripheral blood at 3,000 rpm for 15 minutes at 20°C. Collect 4-5 mL autologous serum for heat-inactivation at 56°C for 30 minutes.
19. Culture enriched primary MM cells in RPMI-1640 medium + 20% heat-inactivated autologous serum (100 µL 0.3x10^6^/mL per well in 96-well plates) at 37°C, 5% CO2.

**Treatment of NSG mice engrafted with OPM2 cells**

Female NSG mice, aged 7-8 weeks, were purchased from The Jackson Laboratory (Bar Harbor, Maine). OPM2 cells, engineered to express Renilla Luciferase reporter and GFP, were suspended in 200 μL of DMEM at a concentration of 2 × 10^6^ cells and injected into the mice via the tail vein. After 3 weeks, engraftment of OPM2 cells was assessed using live imaging with the IVIS Spectrum CT (Perkin Elmer). Mice were then divided into four groups based on luminescence activity. NB73 was administered subcutaneously at a dose of 10 mg/kg, twice weekly, while Venetoclax was administered via oral gavage at a dose of 100 mg/kg, three times weekly. Humane endpoints included paraplegia and weight loss exceeding 10%. The experiment received approval and oversight from the Biomedical Resource Center, Medical College of Wisconsin (IRB approval number AUA6541). Kaplan Meier analysis was used to plot the survive curves and Logrank test with Bonferroni correction for multiple comparisons was performed to calculate p values.

**Patient information and IRB approval**

MM patient specimens were collected between November 2023 and May 2024. Bone marrow and peripheral blood samples were obtained from 8 cases during routine diagnostic procedures at the Second Affiliated Hospital, Shandong University of Traditional Chinese Medicine, after the patients provided signed consent forms. This study was approved by the Medical Ethical Committees of the Second Affiliated Hospital, Shandong University of Traditional Chinese Medicine.
