# Supplementary figures and images for: Synergistic effect of FOXM1 and BCL-2 inhibition in a preclinical treatment study on multiple myeloma
Source: J Transl Med. 2024 Jul 8;22:638. doi: 10.1186/s12967-024-05452-9 (PMC11232139; doi:10.1186/s12967-024-05452-9)

Table S1


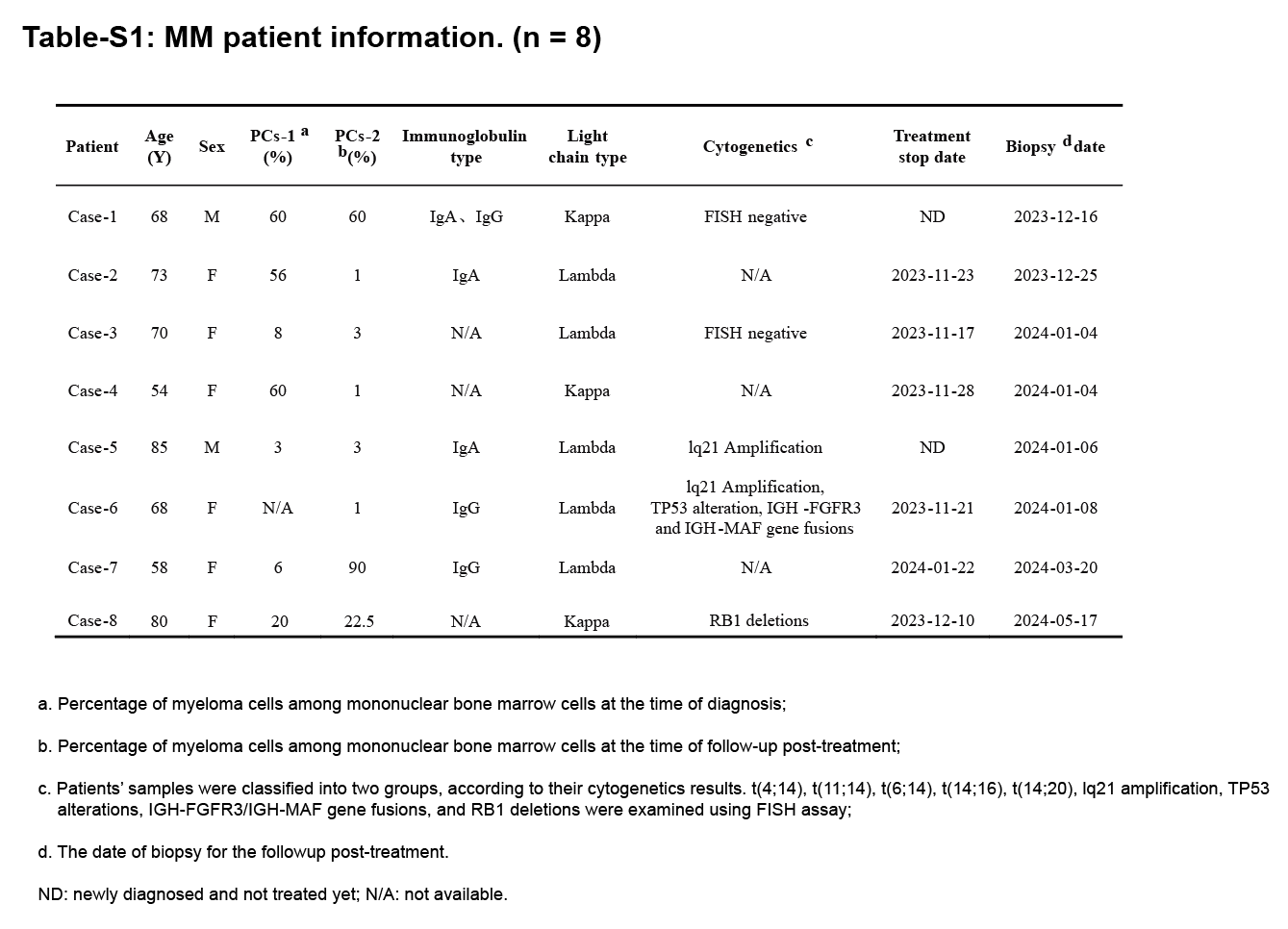

Supplement: Supplementary file 2 — Supplementary Material 2 [file 12967_2024_5452_MOESM2_ESM.docx]
